# Effect of the Application of 1-Methylcyclopropene and Wax Emulsions on Proximate Analysis and Some Antioxidants of Soursop (*Annona muricata* L.)

**DOI:** 10.1155/2014/896853

**Published:** 2014-04-27

**Authors:** Cristina L. Moreno-Hernández, Sonia G. Sáyago-Ayerdi, Hugo S. García-Galindo, Miguel Mata-Montes De Oca, Efigenia Montalvo-González

**Affiliations:** ^1^Laboratorio de Integral de Investigación de Alimentos, Instituto Tecnológico de Tepic, Apartado Postal 634, 63175 Tepic, NAY, Mexico; ^2^UNIDA, Instituto Tecnológico de Veracruz, M. A. de Quevedo 2779, 91897 Veracruz, VER, Mexico

## Abstract

The effect of the application of 1-methylcyclopropene (1-MCP) and wax emulsions, alone or combined, on composition analysis, vitamin C, polyphenols, and antioxidant capacity of soursop was evaluated. Fruits were stored as follows: at 25°C (control), and at 16°C: fruits sprayed with candelilla or flava emulsions, fruits treated with 1500 nL/L of 1-MCP (20°C, 12 h), and fruits treated with 1-MCP and then sprayed with emulsions. Fruits were allowed to ripen and the edible part was used for analysis. Only fruits stored at 16°C without 1-MCP showed visible symptoms of chilling injury. Fruits treated with 1-MCP combined with flava emulsion maintained in greater extent their vitamin C content, dietary fiber, total phenolics content, and antioxidant activity. The combination of 1-MCP and emulsions can be utilized in postharvest handling of soursop because this combination can preserve its nutritional composition and antioxidant activity.

## 1. Introduction

Soursop (*Annona muricata* L.) is a tropical, aromatic, sweet, and great tasting fruit. Mexico is the largest producer in the world and the state of Nayarit is the main producer with 13,022 ton per year. Postharvest losses of soursop of up to 60% have been reported [1]; therefore there is a continuous interest to investigate postharvest treatments that may increase shelf life and maintain the nutritional quality of soursop.

Edible coatings applied on fruits create a semipermeable barrier for respiration gases (O_2_ and CO_2_) and water vapor and consequently reduce both the respiration rate and the ethylene production rate [2]. 1-MCP is commonly employed as an effective inhibitor of ethylene to prevent the cascade of reactions leading to ripening and senescence [3]. In the past few years, technologies have been reported whereby physiological and physicochemical variables during the ripening process of diverse fruits including soursop were evaluated [4–6]. However, to maintain the physiological and physicochemical quality in fruits, it is important that postharvest technologies preserve nutrients such as vitamins, minerals, fiber, and other bioactive compounds. These components contribute to maintain vital functions of the human body and decrease chronic and degenerative processes, such as cardiovascular diseases and cancer [7]. Therefore, in recent years research on the effects of postharvest treatments on nutritional composition and antioxidants content in fruits has been conducted in order to increase their added value.

Changes on bioactive compounds and antioxidant activity as a result of application of edible coatings have not been studied in soursop. However, studies in other fruits such as papaya and mango are available. Adetuyi et al. [8] reported that the use of edible coatings on papaya preserved antioxidants such as polyphenols, tannins, and ascorbic acid during storage. Robles-Sánchez et al. [9] proposed that an alginate-based edible coating maintained color and increased vitamin C content in precut mango cubes. Additionally, there are very few reports on the effect of 1-MCP on nutrients or bioactive compounds. Several studies have demonstrated that 1-MCP reduced the loss of vitamin C in China fruit (*Zizyphus jujuba*) and pineapples because it partly inhibited the activity of oxidative enzymes [10, 11]. The same effect was observed in tomatoes and apples, in which phenolic compounds were found in greater amounts in fruits treated with 1-MCP, with respect to control fruits, by reduction of polyphenoloxidase activity [12]. In this work we studied the effect of 1-methylcyclopropene and application of emulsions, alone or combined, on proximate analysis and some antioxidant compounds in soursop.

## 2. Materials and Methods

1-MCP was a kind donation from AgroFresh Inc. (Philadelphia, PA, USA). Candelilla or flava emulsions were donated from Multiceras S. A. de C. V. (Monterrey Nuevo León, Mexico). All solvents, salts, and acids were purchased from Sigma Chemical Co. (St. Louis, MO) or J. T. Baker (Mexico City). All aqueous solutions were prepared with distilled water.

### 2.1. Plant Material

Soursop fruits were obtained from an orchard located in the village of El Tonino, municipality of Compostela, state of Nayarit, Mexico. Fruits were harvested when their skin was light green; spines were separated and uniform in size. After harvest, fruits were washed and immersed for 5 min in a solution containing 20 mg/L of 2-(4-thiazolyl)-1H-benzimidazole to prevent fungal growth. Fruits were air-dried at 25°C and a relative humidity (RH) of 85–90%.

### 2.2. Treatments

Lots of 20 fruits were used for each of the treatments as follows.

1. Fruits stored at 25°C (Control).
2. Fruits stored at 16°C without 1-MCP (16°C).
3. Fruits treated with 1500 nL/L of 1-MCP (for 12 h at 20°C) and then stored at 16°C (1-MCP).
4. Fruits treated with candelilla or flava emulsions diluted with water 10 : 90 v/v and then stored at 16°C (C10:90 or F10:90).
5. Fruits treated with 1-MCP, immediately applied with emulsions, and then stored at 16°C as mentioned above (1-MCP + C10:90 and 1-MCP + F10:90).

The 1-MCP treatment was applied at 20°C on the same day when fruits were harvested. Exposure to 1-MCP (1500 nL/L diluted in distilled water) was performed in a 225 L sealed chamber fitted with a fan. A 10 mL glass container was used to hold 1-MCP and placed at the bottom of the chamber. The chamber was kept sealed for 12 h and a fan was used to homogenize the release of 1-MCP. Emulsions were diluted with water in a 10 : 90 v/v ratio and were sprayed, alone or after the 1-MCP application, twice on the surface of fruits, and then air-dried.

All fruits were stored during their ripening stage (6 days for fruits stored 25°C, 9 days for fruits stored at 16°C with and without emulsions, 12 and 14 days for fruits stored at 16°C with 1-MCP alone or combined with emulsions), and the edible part or pulp of 20 fruits per treatment was manually extracted and seeds were removed. The pulp was freeze-dried and stored at −75°C until analyzed. Lyophilized pulp was divided into two lots for treatment and analyses were performed in triplicate.

### 2.3. Proximate Analysis

Moisture (Method 925.09), protein (Method 920.87), fat (920.39), and ash (Method 923.03) contents were determined following the official AOAC methods [13]. Soluble sugars were quantified by the phenol-sulfuric method [14]. All data, except moisture (g/100 g fresh weight of edible food, FW), were reported as g/100 g dry matter (DM) of the edible portion.

Total dietary fiber (TDF) is the sum of soluble dietary fiber (SDF) and insoluble dietary fiber (IDF). It was analyzed by the AOAC enzymatic-gravimetric method (Method 991.42) modified by Mañas and Saura-Calixto [15]. Samples were treated with heat stable *α*-amylase, protease, and amyloglucosidase to remove protein and starch. Remaining residues were separated by centrifugation (15 min, 25°C, 3000 ×g) to separate soluble and insoluble fractions. The supernatants were dialyzed with water to avoid losses of SDF. The dialysates (containing SDF) and residues (nonstarch polysaccharides, NSP) obtained from centrifugation were submitted to acid hydrolysis with sulfuric acid (12 M). To calculate SDF and NSP the amount of glucose obtained by hydrolysis was measured using the method of Englyst & Cummings [16]. Remaining residues were quantified gravimetrically as Klason lignin (KL). IDF was calculated as the sum of NSP and KL. Data were reported in g glucose/100 g DM.

### 2.4. Vitamin C

Vitamin C was analyzed using the technique reported by Suntornsuk et al. [17]. Briefly, 10 g of soursop pulp were homogenized with 25 mL of sulfuric acid 2 N, 25 mL of distilled water, and 3 mL of starch solution as indicator. The mixture was titrated with iodine 0.05 N. Data were reported in mg ascorbic acid/100 g FW.

### 2.5. Total Phenolic Content

Total phenolic content is considered as the sum of extractable polyphenols (EP) and nonextractable polyphenols (NEP). EP was extracted by shaking at room temperature with acidic methanol (HCl 0.8%)-water in ratio 50 : 50 (v/v) for 1 h and acetone-water (70 : 30 v/v) for 1 h [18]. After centrifugation (15 min, 25°C, 3000 ×g) supernatants were combined and EP was determined with the Folin-Ciocalteu reagent [19]. Data were reported in gallic acid equivalents (GAE)/100 g DM. NEP is divided in condensed tannins (CT) and hydrolysable polyphenols (HP). Both were measured in the freeze-dried pulp that was extracted with methanol/acetone/water above mentioned. CT was determined in the residue with a hydrolysis treatment by 5 mL/L HCl-Butanol (3 h at 100°C), and then absorbance at 550 nm was measured [20]. CT was calculated with a standard curve from a Mediterranean carob pod (*Ceratonia siliqua *L.) solution. HP were determined by hydrolysis with methanol/H_2_SO_4_ 90 : 10 (v/v) at 85°C for 20 h, on the residues of the methanol/acetone/water extraction described above [21]. Samples were centrifuged (15 min, 25°C, 3000 ×g) and HP was determined in the supernatants by the Folin-Ciocalteu reagent. Data were reported in GAE/100 g DM.

### 2.6. Antioxidant Capacity (AC)

AC was analyzed by ferric reducing antioxidant power (FRAP) assay [22]. It was performed in the supernatants from the methanol/acetone/water extraction described above. Antioxidant capacity was estimated using TPTZ and FeCl_3_, and a standard curve of Trolox was used to estimate the antioxidant capacity of samples expressed as *μ*mol of Trolox equivalents (TE)/100 g DM.

### 2.7. Statistical Analysis

The experiment was performed by duplicate in the same season. Analyses were run in triplicate. The experimental design was completely randomized with a single-factor (postharvest treatment). Data were analyzed by ANOVA using the SAS statistical package (The SAS System for Windows, Version 9.0, SAS Institute, Cary, NC). The LSD test for multiple means comparison was performed. All tests of significance were at 0.05 (*P* < 0.05). Results were expressed as mean ± standard error of the mean.

## 3. Results and Discussion

### 3.1. Proximate Analysis

Table 1 shows that moisture content did not show significant differences between treatments (*P* > 0.05). All samples had moisture contents between 79.0 and 81.0 g/100 g FW. The noticeable variability in moisture of soursop pulp can be attributed to uncontrolled RH during of storage of the fruit until analyzed. Similar values of moisture content for soursop pulp were reported by Onimawo [23].

Protein content in fruits stored at 25°C was 1.03 g/100 g DM, while all refrigerated fruits with or without postharvest treatment had a protein content between 0.69 and 0.73 g/100 g DM, showing lower protein content than control fruits. Astiasaran and Martínez [24] reported that protein content in fruits is composed mainly of metabolic enzymes; hence, protein concentration increased during ripening of fruits because numerous enzymes are synthetized. The low protein content found in the refrigerated fruit pulp may be attributed to decreased metabolic rate caused by the postharvest treatments and refrigerated storage. The soursop fruit showed a reduction in ethylene production rate [25] and probably had a decreased protein synthesis including enzymes [26].

Unlike proteins, the fat content was lower in fruits stored at 25°C than in refrigerated fruits. The loss of fat content in control fruits was due to the softening, which occurs in the cell membrane by the loss of phospholipids during fruit ripening [27]. It could also be attributed to conversion of lipids to volatile compounds in the synthesis of aroma compounds [27]. A study with* Annona cherimola* L. found that weakening of the cellular membrane was associated with the loss of phospholipids. This effect was linked with high ethylene production rate of the fruits during ripening, which led to high enzymatic activity and cell membrane disruption [28]. In all refrigerated fruits (without or with 1-MCP), storage probably caused a decrease in the activity of enzymes that degrade the cell wall and the lipids content did not decrease.

Soluble sugars displayed significant differences between treatments (*P* < 0.05). Control fruits had 74.06 g/100 g DM, while storage at 16°C of soursop fruits, with or without emulsions, generated evident chilling injury symptoms such as browning, hard texture at touch, and abnormal ripening. The low soluble sugar content in the pulp of fruits stored at 16°C was probably caused by the reduction in starch degradation by a low oxidative capacity of the mitochondria [29] and also produced chilling injury symptoms. This coincided with other studies like that by Vishnu Prasanna et al. [30] who reported that the carbohydrate content of cherimoya decreased when fruits were stored at 10–15°C. With respect to 1-MCP, 1-MCP + C10:90, and 1-MCP + F10:90 fruits, chilling injury symptoms were not observed and fruits ripened normally, but soluble sugars were statistically smaller (*P* < 0.05) than in control fruits (25°C) and greater than fruits that were stored at 16°C without 1-MCP. It could be caused by the synergistic effect of 1-MCP and emulsions applied [31].

Ash content in fruits stored at 16°C was significantly different (*P* < 0.05) at all other treatments, and this result coincided with the highest hardness values observed in the fruits. Regarding the greater mineral content in the fruits stored at 16°C with and without emulsions, it can be attributed to the alterations in the structure of cell wall polysaccharides and their covalent cross-links with minerals when they suffer chilling injury, which could maintain higher levels of calcium in the fruit with the formation of calcium pectates [32].

TDF content in Table 2 shows that fruits stored at 25°C had the lowest TDF content with 19.82 g/100 g DM, with 9.31 g/100 g DM of SDF and 10.49 g/100 g DM of IDF. In refrigerated fruits without 1-MCP (16°C, C10:90 and F10:90), TDF values had a range of 20.81–21.66 g/100 g DM, while in the fruits treated with 1-MCP or 1-MCP + emulsions, the results of TDF ranged 22.75–24.17 g/100 g DM, which was significantly higher than for all other treatments. TDF decreased in mature control fruit because of the activity of pectinmethylesterases, polygalacturonases, and cellulases. These enzymes catalyze the deesterification and depolymerization of the polysaccharides that are part of SDF and IDF, such as pectin, hemicellulose, and cellulose in the cell wall [33]. Ramírez and Pacheco [34] reported TDF content in soursop of 49.34 g/100 g DM. However, this difference with respect to our results may be attributed to the analytical method and the soursop variety employed. TDF depended on the SDF and IDF proportion measured for each treatment. However, it was clear that fruits without 1-MCP at 16°C had lower SDF content. This is probably caused by abnormal hardening by chilling injury, which suggests decreased hydrolysis in the cell wall and, consequently, smaller values of SDF and greater values of IDF. Fruits treated with 1-MCP and 1-MCP + emulsions had greater amounts of TDF than control fruit and refrigerated fruit without 1-MCP. It is possible that these fruits had an increase in SDF caused by hydrolysis of cell wall material given that normally fruits were soft, but the hydrolysis was not in the same extent than control fruit, because the latter showed a higher content of IDF caused by the application of 1-MCP and edible coatings. These results coincided with firmness retention in fruits treated with 1-MCP and emulsions [6]. It has also been reported that either together or in separate applications 1-MCP and edible coatings can achieve the reduction in the activity of hydrolases or fruit softening [5, 35, 36].

### 3.2. Vitamin C

**Figure 1:**
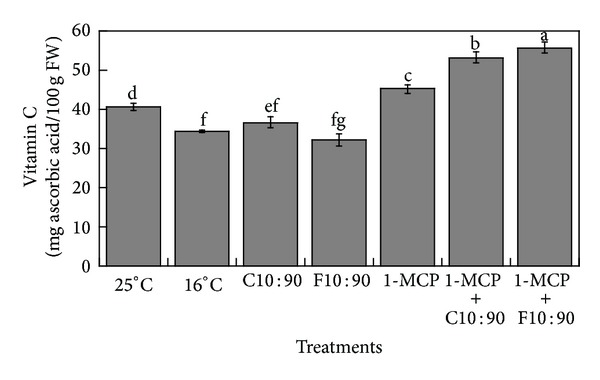
Vitamin C (mg ascorbic acid/100 g fresh weight of edible food) of seedless pulp obtained from soursop stored at 25°C, 16°C, fruits with emulsions and stored at 16°C; fruits treated with 1-MCP and stored at 16°C; fruits treated with 1-MCP + emulsions and stored at 16°C. Different letters indicate significant statistical differences between samples using the LSD test with *α* = 0.05.

Vitamin C content in control fruit (25°C) was 40.56 mg/100 g FW (Figure 1). Fruits stored at 16°C, with or without emulsions had a statistically smaller (*P* < 0.05) vitamin C content than control fruits (25°C). In fruits treated with 1-MCP we found values of 45.24 mg/100 g FW; meanwhile in 1-MCP + C10:90 and 1-MCP + F10:90 fruits, vitamin C content values were 53.30 and 55.78 mg/100 g FW, respectively. The vitamin C content in control fruits was high even if compared to values reported by Barreca et al. [7] in soursop, who found a concentration of 3.30 mg/100 g FW. Fruit varieties employed and environmental conditions could explain the difference. In fruits stored at 16°C without 1-MCP it is possible that chilling injury contributed to reduce the synthesis of vitamin C; while in fruits stored at 16°C with 1-MCP, this compound could prevent chilling injury and at the same time delay the synthesis of enzymes that degrade ascorbic acid and also edible coatings acted synergistically with 1-MCP to reduce gas exchange and decrease the oxidation of vitamin C [37, 38]. Ma et al. [39] reported that the application of 1-MCP in broccoli decreased the gene expression of ascorbate peroxidase precursor synthesis, the main oxidative enzyme of ascorbic acid. Qiuping and Wenshui [40] found that the combined treatment with 1-MCP and chitosan in China fruit (*Ziziphus mauritiana*) showed better retention of vitamin C than control fruit. Also, Sivakumar et al. [41] reported the highest vitamin C content in mango fruit treated with 1-MCP with respect to control fruit. It is possible that the ascorbate peroxidase activity decreased and ascorbic acid content was retained in soursop; however, more research is needed to elucidate the roles of enzymes and precursors.

### 3.3. Total Phenolic Content

The effect of postharvest treatments was significant (*P* < 0.05) for total phenolic content (Table 3). Control fruits showed a total phenolic content of 2.55 g GAE/100 g DM; however, in all fruits stored at 16°C the amount of total polyphenols was higher than control fruit. In fruits maintained at 16°C, we found 2.61 g GAE/100 g DM while in C10:90 and F10:90 fruits the CT and HP content was slightly higher than fruits at 16°C; therefore, total phenolic content was 2.91–2.93 g GAE/100 g DM. Fruits with 1-MCP and 1-MCP + emulsions application retained higher values of total phenolics in all treatments; however the 1-MCP + emulsions (candelilla or flava) treatments preserved the highest content of EP, CT, and HP and at the same time increased shelf life. Barreca et al. [7] reported a polyphenol content of 0.054 g GAE/100 g DM in soursop, which did not coincide with the data of total polyphenolic content found in our experiments for control fruits, because the former authors quantified EP only. The lowest content of EP and HP in fruits at 16°C with and without emulsions was related to intense browning in the pulp caused by chilling injury. Lima de Olivera et al. [42] reported that this physiopathy in soursop occurred by the oxidation of phenolic compounds because of polyphenoloxidase (PPO) activity. Adetuyi et al. [43] found that the decrease of tannins during ripening of orange fruit was caused by PPO, which turned tannins into simple phenols. The use of emulsions could reduce the exchange of oxygen and thus produce lower PPO activity and preserve tannins since fruits with emulsions had higher contents of CT. Waxes were used on lychee and pomegranate as a postharvest application and it was found that PPO activity decreased. Also, in these same fruits was found greater content of phenols and tannins than control fruit [38, 44]. It has been reported that application of 1-MCP decreased PPO activity and preserved EP [45]. The effect of 1-MCP and emulsion combined applications on HP and CT has not been studied; however it is possible that both treatments act synergistically to decrease the synthesis and activity of enzymes that degrade these compounds.

### 3.4. Antioxidant Capacity (AC)

The AC in control fruit was 76.07 *μ*mol TE/g DM (Table 3), while AC in fruits stored at 16°C with or without emulsion AC was lower (72.29–75.65 *μ*mol TE/g DM). AC increased in fruits treated with 1-MCP + emulsions and was highest in 1-MCP + F10:90 (89.84 *μ*mol TE/g DM). 1-MCP and 1-MCP + emulsion treatments preserved vitamin C and total polyphenols in fruits; therefore greater AC was possible [46]. The EP of each treatment showed a high correlation (*r* ^2^ = 0.9606 − 0.9704) with the AC, indicating that the EP contained in soursop had the ability to reduce metals such as iron (Fe); however vitamin C showed a low correlation (*r* ^2^ = 0.4969 − 0.4956). The correlations found between PE and AC agree with reports by other authors [47]. High AC is likely because EP have a large number of hydroxyl (OH) functional groups in their chemical structure, which are responsible for conferring its high antioxidant power. Gil et al. [48] found no correlation between vitamin C and the AC determined by the FRAP or ABTS assays in nectarines, peaches, and plums. Thus we propose that the AC of vitamin C is largely dependent on the amount originally present in the fruit.

## 4. Conclusions

The combined treatment with 1-MCP and flava emulsion was more effective to preserve high content of dietary fiber, total polyphenols, vitamin C, and antioxidant capacity in soursop. The results found in this experiment suggest that the combination of 1-MCP with emulsions can be an alternative to preserve the nutritional value of soursop and increase its shelf life.

## Figures and Tables

**Table 1:** Proximate analysis of seedless pulp obtained from soursop stored at 25°C, 16°C, fruits with emulsions and stored at 16°C; fruits treated with 1-MCP and stored at 16°C; fruits treated with 1-MCP + emulsions and stored at 16°C (g/100 g dry matter of edible portion)*.

Treatment	^†^Moisture	Protein	Fat	Soluble sugars	Ash
25°C (control)	80.71 ± 0.82^a^	1.03 ± 0.01^a^	0.77 ± 0.07^c^	74.06 ± 1.30^a^	3.32 ± 0.03^bc^
16°C	81.16 ± 2.36^a^	0.73 ± 0.07^b^	1.37 ± 0.08^a^	69.37 ± 0.41^c^	3.86 ± 0.16^a^
C10:90	80.55 ± 1.01^a^	0.69 ± 0.06^b^	1.33 ± 0.04^ab^	69.51 ± 0.16^c^	3.47 ± 0.09^b^
F10:90	80.40 ± 1.84^a^	0.71 ± 0.05^b^	1.35 ± 0.09^ab^	68.53 ± 0.60^c^	3.16 ± 0.13^bc^
1-MCP	79.83 ± 1.28^a^	0.75 ± 0.01^b^	1.15 ± 0.01^bc^	71.47 ± 0.52^b^	3.14 ± 0.13^bc^
1-MCP + C10:90	79.69 ± 0.19^a^	0.72 ± 0.08^b^	1.22 ± 0.08^b^	71.26 ± 0.23^b^	3.00 ± 0.19^c^
1-MCP + F10:90	80.55 ± 1.01^a^	0.72 ± 0.04^b^	1.28 ± 0.16^b^	70.02 ± 1.05^b^	2.96 ± 0.12^d^

*Values are the mean ± SD (*n* ≥ 6). Different letters in columns indicate significant differences between samples using LSD test with *α* = 0.05. ^†^Moisture is reported in g/100 g fresh weight of edible food.

**Table 2:** Soluble (SDF), insoluble (IDF), and total dietary fiber (TDF) of seedless pulp obtained from soursop stored at 25°C, 16°C, fruits with emulsions and stored at 16°C; fruits treated with 1-MCP and stored at 16°C; fruits treated with 1-MCP + emulsions and stored at 16°C (g glucose/100 g dry matter of edible portion)*.

Treatment	SDF	IDF		TDF
		^†^NSP	Klason lignin	
25°C (control)	9.31 ± 0.96^ab^	4.93 ± 0.69^d^	5.56 ± 0.48^a^	19.82 ± 0.24^c^
16°C	8.69 ± 0.27^c^	6.64 ± 0.31^c^	5.31 ± 0.41^ab^	21.66 ± 0.47^b^
C10:90	8.31 ± 0.26^c^	8.51 ± 0.29^b^	4.32 ± 0.70^bc^	20.81 ± 0.61^bc^
F10:90	8.47 ± 0.95^c^	8.45 ± 0.40^b^	4.02 ± 0.77^c^	21.45 ± 1.32^b^
1-MCP	9.77 ± 0.11^ab^	7.31 ± 0.33^bc^	5.66 ± 0.10^a^	22.75 ± 0.15^b^
1-MCP + C10:90	8.97 ± 0.39^b^	8.75 ± 0.13^a^	4.79 ± 0.19^b^	23.80 ± 0.35^a^
1-MCP + F10:90	10.25 ± 0.08^a^	8.83 ± 0.40^a^	4.13 ± 0.10^c^	24.17 ± 0.98^a^

*Values are the mean ± SD (*n* ≥ 6). Different letters in columns indicate significant differences between samples using LSD test with *α* = 0.05. ^†^Nonstarch polysaccharides.

**Table 3:** Extractable polyphenols (EP), hydrolysable polyphenols (HP), condensed tannins (CT), total phenolic content (TPC) (GAE/100 g dry matter of edible food), and ferric reducing antioxidant power (FRAP, µmol ET/g dry matter of edible food) of seedless pulp obtained from soursop stored at 25°C, 16°C, fruits with emulsions and stored at 16°C; fruits treated with 1-MCP and stored at 16°C; fruits treated with 1-MCP + emulsions and stored at 16°C.

Treatment	EP	HP	CT	TPC	FRAP
25°C (control)	*1.14 ± 0.04^b^	0.25 ± 0.01^c^	1.16 ± 0.03^e^	2.55 ± 0.02^d^	76.07 ± 1.08^b^
16°C	0.99 ± 0.01^c^	0.28 ± 0.01^c^	1.34 ± 0.13^d^	2.61 ± 0.05^c^	72.29 ± 1.03^c^
C10:90	1.05 ± 0.01^c^	0.37 ± 0.01^b^	1.51 ± 0.12^b^	2.93 ± 0.04^b^	74.59 ± 2.82^bc^
F10:90	1.00 ± 0.05^c^	0.34 ± 0.07^bc^	1.57 ± 0.03^b^	2.91 ± 0.05^b^	75.65 ± 1.36^b^
1-MCP	1.00 ± 0.03^c^	0.40 ± 0.60^ab^	1.59 ± 0.02^b^	2.99 ± 0.55^ab^	83.50 ± 0.47^a^
1-MCP + C10:90	1.37 ± 0.04^a^	0.42 ± 0.09^a^	1.83 ± 0.03^a^	3.62 ± 0.05^a^	87.37 ± 9.67^a^
1-MCP + F10:90	1.29 ± 0.07^a^	0.44 ± 0.06^a^	1.79 ± 0.03^a^	3.52 ± 0.04^a^	89.84 ± 0.72^a^

*Values are the mean ± SD (*n* ≥ 6). Different letters in columns indicate significant differences between samples using LSD test with *α* = 0.05.

## References

[1] SAGARPA (2013). *Secretaria de Agricultura, Ganadería, Desarrollo Rural, Pesca y Alimentación*.

[2] Falguera V, Quintero JP, Jiménez A, Muñoz JA, Ibarz A (2011). Edible films and coatings: structures, active functions and trends in their use. *Trends in Food Science and Technology*.

[3] Blankenship SM, Dole JM (2003). 1-Methylcyclopropene: a review. *Postharvest Biology and Technology*.

[4] Jeong J, Huber DJ, Sargent SA (2003). Delay of avocado (*Persea americana*) fruit ripening by 1-methylcyclopropene and wax treatments. *Postharvest Biology and Technology*.

[5] de Lima MAC, Alves RE, Filgueiras HAC (2010). Respiratory behavior and softening of soursop fruit (*Annona muricata* L.) after postharvest treatments with wax and 1-methylcyclopropene. *Ciencia e Agrotecnologia*.

[6] Tovar-Gómez B, Mata-Montes De Oca M, García-Galindo HS, Montalvo-González E (2011). Efecto de emulsiones de cera y 1-metilciclopropeno en la conservación poscosecha de guanabana. *Revista Chapingo Serie Horticultura*.

[7] Barreca D, Laganà G, Ficarra S (2011). Evaluation of the antioxidant and cytoprotective properties of the exotic fruit *Annona cherimola* Mill. (Annonaceae). *Food Research International*.

[8] Adetuyi FO, Akinadewo LT, Omosuli SV, Ajala L (2008). Antinutrient and antioxidant quality of waxed and unwaxed pawpaw *Carica papaya* fruit stored at different temperatures. *African Journal of Biotechnology*.

[9] Robles-Sánchez MR, Rojas-Graü MA, Odriozola-Serrano I, González-Aguilar G, Martin-Belloso O (2013). Influence of alginate-based edible coating as carrier of antibrowning agents on bioactive compounds and antioxidant activity in fresh-cut Kent mangoes. *LWT—Food Science and Technology*.

[10] Jiang W, Sheng Q, Jiang Y, Zhou X (2004). Effects of 1-methylcyclopropene and gibberellic acid on ripening of Chinese jujube (*Zizyphus jujuba* M) in relation to quality. *Journal of the Science of Food and Agriculture*.

[11] Selvarajah S, Bauchot AD, John P (2001). Internal browning in cold-stored pineapples is suppressed by a postharvest application of 1-methylcyclopropene. *Postharvest Biology and Technology*.

[12] Wang M, Cao J, Lin L, Sun J, Jiang W (2010). Effect of 1-methylcyclopropene on nutritional quality and antioxidant activity of tomato fruit (*Solanum lycopersicon* L.) during storage. *Journal of Food Quality*.

[13] AOAC (1990). *Official Methods of Analysis of Association Official of Agricultural Chemists International*.

[14] Dubois M, Gilles KA, Hamilton JK, Rebers PA, Smith F (1956). Colorimetric method for determination of sugars and related substances. *Analytical Chemistry*.

[15] Mañas E, Saura-Calixto F (1995). Dietary fibre analysis: methodological error sources. *European Journal of Clinical Nutrition*.

[16] Englyst HN, Cummings JH (1988). Improved method for measurement of dietary fiber as non-starch polysaccharides in plant foods. *Journal of the Association of Official Analytical Chemists*.

[17] Suntornsuk L, Gritsanapun W, Nilkamhank S, Paochom A (2002). Quantitation of vitamin C content in herbal juice using direct titration. *Journal of Pharmaceutical and Biomedical Analysis*.

[18] Pérez-Jiménez J, Arranz S, Tabernero M (2008). Updated methodology to determine antioxidant capacity in plant foods, oils and beverages: extraction, measurement and expression of results. *Food Research International*.

[19] Montreau FR (1972). Sur le dosage des composés phénoliques totaux dans les vins par la methode Folin-Ciocalteau. *Connaissance de la Vigne et Du Vin*.

[20] Reed JD, Mcdowell RT, Van Soest PJ, Horvath PR (1982). Condensed tannins: a factor limiting the use of cassava forage. *Journal of the Science of Food and Agriculture*.

[21] Hartzfeld PW, Forkner R, Hunter MD, Hagerman AE (2002). Determination of hydrolyzable tannins (gallotannins and ellagitannins) after reaction with potassium iodate. *Journal of Agricultural and Food Chemistry*.

[22] Benzie IFF, Strain JJ (1996). The ferric reducing ability of plasma (FRAP) as a measure of “antioxidant power”: the FRAP assay. *Analytical Biochemistry*.

[23] Onimawo IA (2002). Proximate composition and selected physicochemical properties of the seed, pulp and oil of sour sop (*Annona muricata*). *Plant Foods for Human Nutrition*.

[24] Astiasaran I, Martínez J (2000). *Alimentos: Composición y Propiedades*.

[25] Espinosa I, Ortiz R, Tovar B, Mata M, Montalvo E (2013). Physiological and physicochemical behavior of soursop fruits refrigerated with 1-methylcyclopropene. *Journal of Food Quality*.

[26] Hagenmaier RD (2005). A comparison of ethane, ethylene and CO_2_ peel permeance for fruit with different coatings. *Postharvest Biology and Technology*.

[27] Knee M (2002). *Fruit Quality and Its Biological Basis*.

[28] Gutiérrez M, Mar Sola M, Vargas AM (2005). Fatty acid composition of phospholipids in mesocarp of cherimoya fruit during ripening. *Food Chemistry*.

[29] Kader AA (1992). Postharvets biology and technology: an overview. *Tehnology of Agricultural Crops*.

[30] Vishnu Prasanna KN, Sudhakar Rao DV, Krishnamurthy S (2000). Effect of storage temperature on ripening and quality of custard apple (*Annona squamosa* L.) fruits. *Journal of Horticultural Science and Biotechnology*.

[31] Watkins CB (2006). The use of 1-methylcyclopropene (1-MCP) on fruits and vegetables. *Biotechnology Advances*.

[32] Maldonado R, Molina-Garcia AD, Sanchez-Ballesta MT, Escribano MI, Merodio C (2002). High CO_2_ atmosphere modulating the phenolic response associated with cell adhesion and hardening of *Annona cherimola* fruit stored at chilling temperature. *Journal of Agricultural and Food Chemistry*.

[33] Cheng G, Duan X, Jiang Y (2009). Modification of hemicellulose polysaccharides during ripening of postharvest banana fruit. *Food Chemistry*.

[34] Ramírez A, Pacheco E (2011). Composición química y compuestos bioactivos presentes en pulpas de piña, guayaba y guanábana. *Interciencia*.

[35] Zhou R, Li Y, Yan L, Xie J (2011). Effect of edible coatings on enzymes, cell-membrane integrity, and cell-wall constituents in relation to brittleness and firmness of Huanghua pears (*Pyrus pyrifolia* Nakai, cv. Huanghua) during storage. *Food Chemistry*.

[36] Krongyut W, Srilaong V, Uthairatanakij A, Wongs-Aree C, Esguerra EB, Kanlayanarat S (2011). Physiological changes and cell wall degradation in papaya fruits cv. “Kaek Dum” and “Red Maradol” treated with 1- methylcyclopropene. *International Food Research Journal*.

[37] Valdenegro M, Fuentes L, Herrera R, Moya-León MA (2012). Changes in antioxidant capacity during development and ripening of goldenberry (*Physalis peruviana* L.) fruit and in response to 1-methylcyclopropene treatment. *Postharvest Biology and Technology*.

[38] Barman K, Asrey R, Pal RK, Kaur C, Jha SK (2014). Influence of putrescine and carnauba wax on functional and sensory quality of pomegranate (*Punica granatum* L.) fruits during storage. *Journal of Food Science and Technology*.

[39] Ma G, Zhang L, Kato M (2010). Effect of 1-methylcyclopropene on the expression of genes for ascorbate metabolism in postharvest broccoli. *Postharvest Biology and Technology*.

[40] Qiuping Z, Wenshui X (2007). Effect of 1-methylcyclopropene and/or chitosan coating treatments on storage life and quality maintenance of Indian jujube fruit. *LWT—Food Science and Technology*.

[41] Sivakumar D, Van Deventer F, Terry LA, Polenta GA, Korsten L (2012). Combination of 1-methylcyclopropene treatment and controlled atmosphere storage retains overall fruit quality and bioactive compounds in mango. *Journal of the Science of Food and Agriculture*.

[42] Lima de Olivera S, Barbosa GN, Sucupira MI, Souza LAV (1994). Polyphenoloxidase activity, polyphenols concentration and browning intensity during soursop (*Annona muricata* L.) maturation. *Journal of Food Science*.

[43] Adetuyi FO, Ibrahim TA, Ajalia L, Oloye DA (2010). Waxing effect on the physical attributes, antioxidant and sugar contents of orange (Citrus sinensis L. osbeek) stored at room temperature in Nigeria. *Bangladesh Journal of Scientific and Industrial Research*.

[44] Dong H, Cheng L, Tan J, Zheng K, Jiang Y (2004). Effects of chitosan coating on quality and shelf life of peeled litchi fruit. *Journal of Food Engineering*.

[45] Lee J, Cheng L, Rudell DR, Watkins CB (2012). Antioxidant metabolism of 1-methylcyclopropene (1-MCP) treated “Empire” apples during controlled atmosphere storage. *Postharvest Biology and Technology*.

[46] Tan CK, Ali ZM, Ismail I, Zainal Z (2012). Effects of 1-methylcyclopropene and modified atmosphere packaging on the antioxidant capacity in Pepper “Kulai” during low-temperature storage. *The Scientific World Journal*.

[47] Thaipong K, Boonprakob U, Crosby K, Cisneros-Zevallos L, Hawkins Byrne D (2006). Comparison of ABTS, DPPH, FRAP, and ORAC assays for estimating antioxidant activity from guava fruit extracts. *Journal of Food Composition and Analysis*.

[48] Gil MI, Tomás-Barberán FA, Hess-Pierce B, Kader AA (2002). Antioxidant capacities, phenolic compounds, carotenoids, and vitamin C contents of nectarine, peach, and plum cultivars from California. *Journal of Agricultural and Food Chemistry*.

